# Phylogeography of the Sino-Himalayan Fern *Lepisorus clathratus* on “The Roof of the World”

**DOI:** 10.1371/journal.pone.0025896

**Published:** 2011-09-30

**Authors:** Li Wang, Zhi-Qiang Wu, Nadia Bystriakova, Stephen W. Ansell, Qiao-Ping Xiang, Jochen Heinrichs, Harald Schneider, Xian-Chun Zhang

**Affiliations:** 1 State Key Laboratory of Systematic and Evolutionary Botany, Institute of Botany, The Chinese Academy of Sciences, Beijing, China; 2 Albrecht-von-Haller Institute of Plant Sciences, Georg-August University Göttingen, Göttingen, Germany; 3 Department of Botany, The Natural History Museum London, London, United Kingdom; 4 Graduate University of Chinese Academy of Sciences, Beijing, China; Montreal Botanical Garden, Canada

## Abstract

**Background:**

The Qinghai-Tibetan Plateau (QTP) and its southern and southeastern mountain ranges, Himalaya-Hengduan Mountains (HHM), are one of the most extensive habitats for alpine plants in the world. How ferns occurring in QTP and HHM changed their distribution ranges in response to Quaternary climatic oscillations remains almost unknown.

**Methodology and Results:**

We employed sequences of two chloroplast DNA regions, *rps*4-*trn*S and *trn*L-*trn*F, to reconstruct phylogeography of the Sino-Himalayan fern *Lepisorus clathratus*, occurring mainly in the QTP and HHM. Individuals of this species have either dehiscent or indehiscent sporangia with the latter evolved from the plesiomorphic dehiscent forms. Based on a range-wide sampling, we detected 27 cpDNA haplotypes that were divided into five groups by network analyses. Populations in the Hengduan Mountains possess the highest genetic diversity, while a single haplogroup is detected across the north-central region. A distinct phylogeographical subdivision was detected between the Hengduan Mountains and north-central region by AMOVA analysis. The haplogroup distribution pattern, coalescence and AMOVA analysis suggest that a long term survival area (refugia) of the species was located in the Hengduan Mountains during glaciations, with probable range expansions into north-central regions during interglacial periods. Populations with indehiscent sporangium can carry private haplotypes and are inclined to maintain genetic homogeneity. One group with indehiscent sporangia most likely survived in situ on the QTP during glaciations.

**Conclusions/Significance:**

This study for the first time sheds light on the response of alpine ferns in the QTP and HHM to the Quaternary climatic oscillations.

## Introduction

The Himalaya-Hengduan Mountains region (HMM) is home to over 20,000 species of vascular plants and harbors the richest alpine flora on earth, with notable richness of endemic species [1], [2]. The region extends along the southern frontier to the southeastern rim of the Qinghai-Tibetan Plateau (QTP), which comprises an area of approximately 2.5×10^6^ km^2^ with average altitudes of ca. 4,000 m. It is commonly accepted that the QTP uplift had a major impact on the development of the Asian climate system and on East Asian biodiversity [3], [4]. The vegetation in the QTP and HHM is regarded as highly sensitive and vulnerable to climate change [5]. This vulnerability may be reflected in the genetic pattern of extant populations/species occurring in this region because climatic oscillation of the Pleistocene has likely shaped the spatial distribution of genetic diversity [6].

Phylogeographical analyses are now widely used to reconstruct the history of species using genealogical data within and between populations [7]. However, our knowledge of phylogeographical histories of organisms occurring in the QTP and HHM as well as causal correlations with climatic fluctuations has been limited so far, due to finite phylogeographical studies in the QTP and its adjacent areas in particular for plants [8]–[18]. Interestingly, these studies suggest remarkable differences in the phylogeographical history of the species involved, although two main patterns might be inferred. The first common pattern is found in species that probably survived in refugia on the eastern edge of the central plateau during the interglacial and postglacial periods, such as *Pedicularis longiflora* [12] and *Juniperus przewalskii* [9]. The second common pattern is found in cold-tolerant species, such as *Potentilla fruticosa* [18] and *Aconitum gymnandrum* [13], which may have survived in high-altitude parts of the plateau during the Last Glacial Maximum (LGM). These taxa may have maintained their range perhaps also during previous Pleistocene glaciations, in which no massive ice sheets were developed on the QTP [19]. Unfortunately, most previous studies have sampled only in the QTP and HHM, and the postglacial recovery of northern China has been addressed by only a few studies [11]. Furthermore, all phylogeographical plant studies have focused on seed plants, and until now no research concerning phylogeographical patterns and processes has been carried out for other land plants occurring in this region, such as ferns. Ferns not only contribute a significant number of species to the biodiversity hotspots in SE Asia [20], but also they are likely well suited for reconstructing the impact of climatic oscillations on the spatial distribution of biodiversity.

Here, we selected the alpine fern *Lepisorus clathratus* (C. B. Clarke) Ching (Polypodiacae) to explore the phylogeographical history of alpine ferns in the QTP - HHM mountain systems. This species is a common fern in the HHM, stretching eastward to Northern China (Beijing and Inner Mongolia) and Japan, northward to South Siberia (Russian Altai Mountains), and westward along the Indo-Himalaya through Bhutan, Sikkim, Darjeeling, Nepal to West Indo-Himalaya (Himachal Pradesh; Jammu and Kashmir; Pakistan). The species is also one of the most widely distributed plant taxa in the QTP. The species grows in fissures and crevices of natural rock outcrops and shows a preference for alpine habitats, from ca. 2,000 m up to 5,000 m. The taxonomy in this monophyletic complex has been the subject of controversial discussions [21]–[26] because of some morphological variation and the unusual occurrence of two types of sporangia. Species number has been contentious, from over 20 [21] to 5 [25], and some researchers even treated it as a single species, *L. clathratus* [27]–[29]. Two sporangial types (dehiscent and indehiscent) have been documented in this monophylum. Individuals with dehiscent sporangia (regular type) occur throughout the range of the species, while representatives with indehiscent sporangia (irregular type) are restricted to the QTP and HHM. These specimens were previously treated as an independent genus *Platygyria* Ching [24], [30], which was rejected in our phylogenetic study of *Lepisorus* [31], as *Platygyria* was nested within *Lepisorous clathratus*. The results are consistent with the interpretation of the morphological evidence by C. Fraser-Jenkins [27]–[29] in which *L. clathratus* was considered as the correct species name for both specimens with regular and irregular sporangia. Here, we accept the broader concept of *Lepisorus clathratus* because the single-species concept appears to fit best with the observed data [31]. The present study adds new evidence to the question about the number of species but further studies using nuclear markers are required to confirm the proposal of a single species versus alternative proposals accepting up to 5 species [25].

In this study, we used sequences of chloroplast genome (cpDNA) to investigate the phylogeographical pattern of *L. clathratus* based on a range-wide sampling. Our study aims to address the following questions: (1) does the pattern of cpDNA variation of wide-spread entities in the north-central region relate to the genetic variation found in QTP or HHM regions? (2) Where were the glacial refugia for entities occurring on the QTP during glacitations? Was there a single eastern plateau edge refugium, or did multiple microrefugia exist, possibly including some on the QTP itself? Is there any difference between the phylogeographical pattern in ferns and that in studied angiosperms? (3) Do the sporangial types correlate with spatial genetic differentiation and affect population structure? Indehiscent sporangia restrict the dispersal capacity and thus populations with indehiscent sporangia may show significant different population structures compared with populations with dehiscent sporangia.

## Results

### Sequence variation and relationships among haplotypes

By examining 1,655 bp of the combined dataset including the chloroplast DNA region *rps*4*-trn*S and *trn*L*-trn*F, we detected 27 haplotypes (H1 - H27), which clustered into five main haplotype groups (G1 – G5). Variable sites among these haplotypes were observed as 29 nucleotide substitutions, of which 17 were parsimony informative.

**Figure 1:**
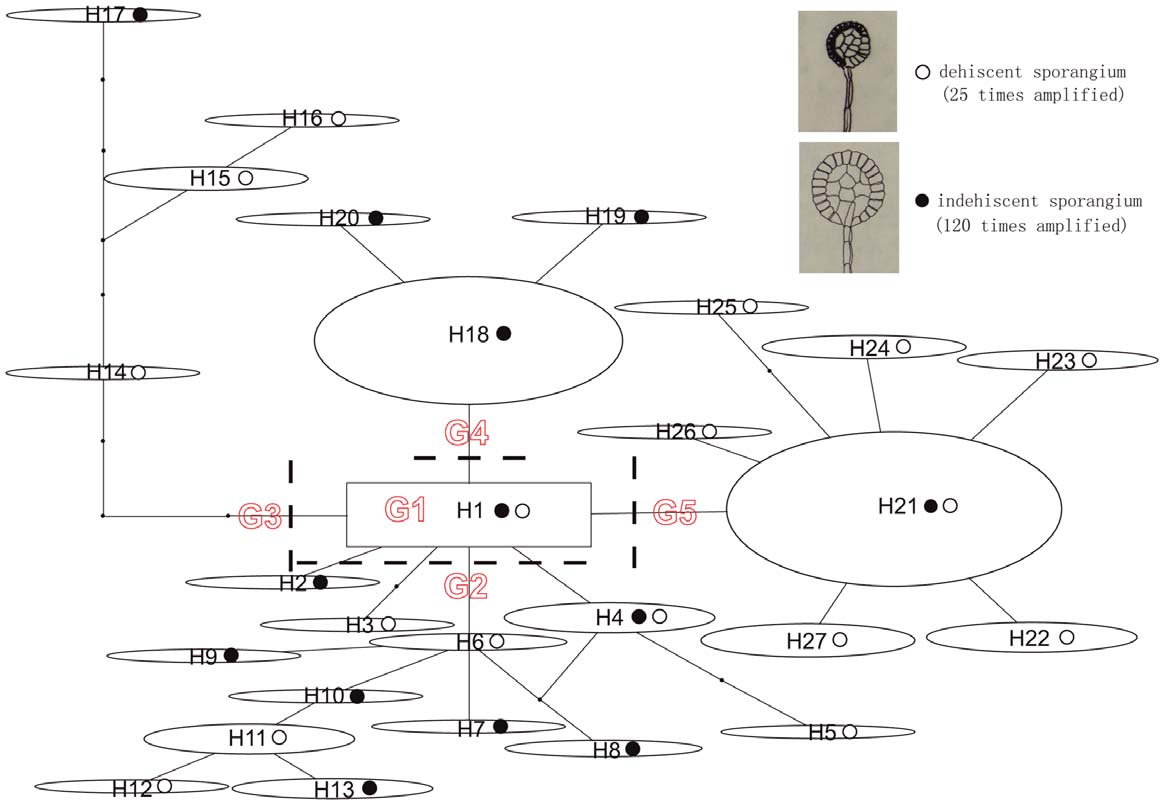
Network of the 27 cpDNA haplotypes detected from *trn*L-F and *rps*4-*trn*S of *L. clathratus*. The bold black dashed lines separate these haplotypes into five groups (G1 – G5). The relative sizes of the ellipses in the network are proportional to haplotype frequencies and black dots represent missing haplotypes (not sampled or extinct). Occurrence of sporangium types in each haplotype is indicated with black (indehiscent sporangium) and white (dehiscent sporangium) circles. Images of two sporangial types are given in the top-right corner.

Relationships between the *L. clathratus* chloroplast haplotypes are shown as a statistical parsimony network in Fig. 1. Haplotype 1 (G1) was located in the center of the network, and the other main haplogroups were connected to it. The other haplotypes are divided into the other four groups (Fig. 1). G2 comprises haplotypes H2 – H13, which are all rare haplotypes occurring in a single or very few individuals (Table S1). Haplotypes H14 – H17 constitute G3, and are well isolated from the central haplotype H1 by at least 4 mutational steps, which is a rather long mutation pathway in comparison to those linking other haplogroups (Fig. 1). G4 is one mutational step from G1 and consists of haplotypes H18 – H20. H19 and H20 are one mutational step derived from H18, the most common haplotype for individuals with indehiscent sporangia. G5 includes haplotypes H21 – H27. Tip haplotypes H22 – H27 are each confined to a few individuals and are derived from H21, the most frequent haplotype among dehiscent individuals. Specimens with indehiscent sporangia were found in each of the haplotype groups (see open and close dotes in Fig. 1).

### Geographical distribution of genetic variation

**Figure 2:**
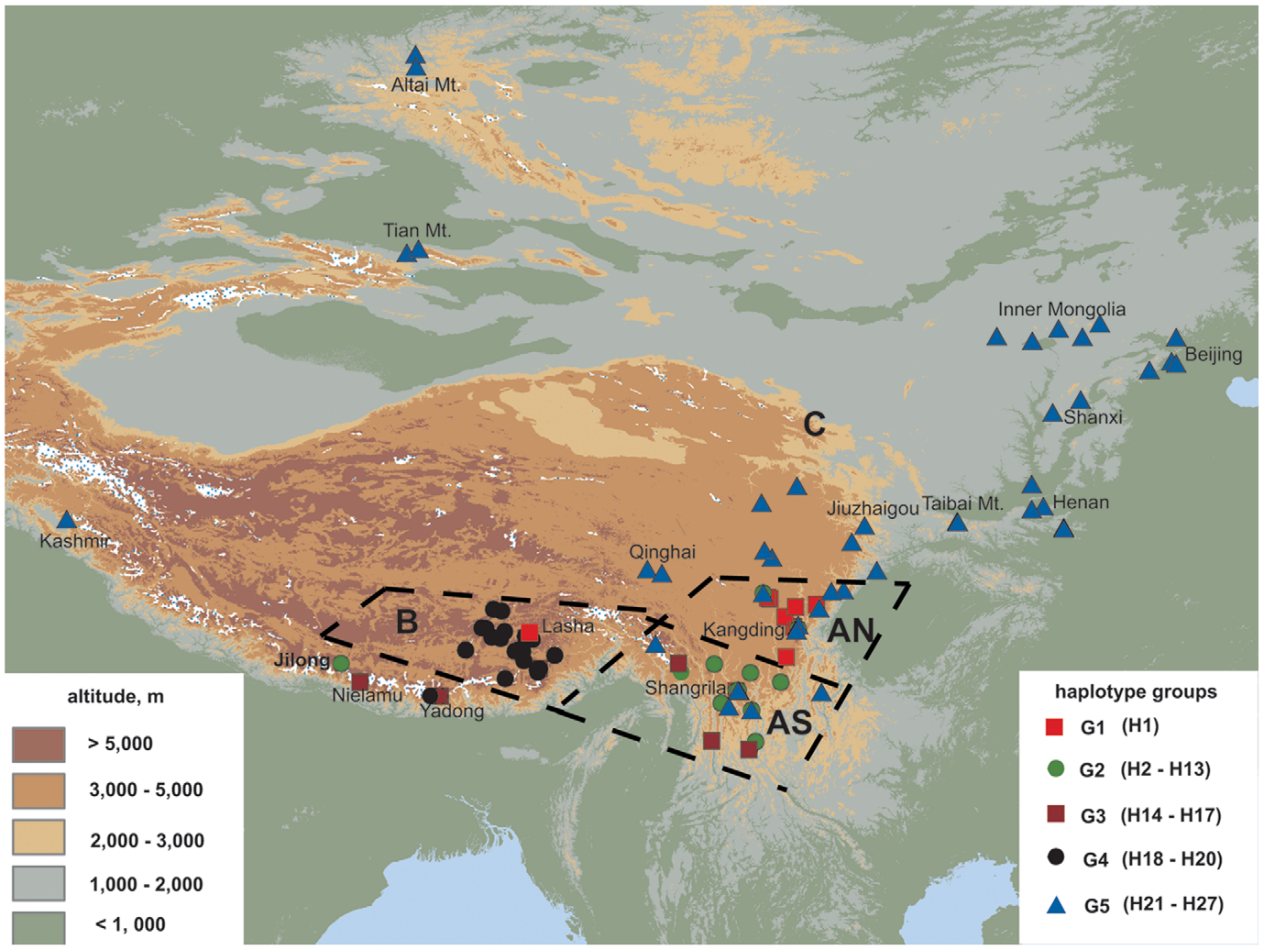
The distribution map of cpDNA haplogroups in *L. clathratus*. Various colors are applied to indicate different altitudinal gradients. The names of some key localities are shown. The bold black dashed lines separate the whole distribution range into three big regions: Hengduan Mountains (A), the QTP (B) and north-central China plus the Altai and Kashmir (C). Region A is divided into two sub-regions, North Hengduan Mountains (AN) and South Hengduan Mountains (AS).

G1 haplogroup was mostly distributed in SW Sichuan, except for two individuals occurring in NW Yunnan (Shangri-La/Zhongdian) and the QTP (Mozhugongka) respectively (Fig. 2). Conversely, G2 was mainly distributed in Yunnan with few specimens extending its range to southern Himalaya (the south slope of the Himalaya), the QTP and SW Sichuan. The genetically long-distance isolated haplogroup G3 tends to occur at the southern fringe of the distribution range of the species, such as the southern Himalaya. The common haplogroup for indehiscent specimens G4 was restricted to the QTP, with only one individual occurring outside this area in the southern Himalaya (Yadong). The most common haplogroup G5 also had the widest distribution, from Yunnan to north-central China, the Russian Altai mountains and Kashmir.

The frequencies and distributions of each haplogroup and molecular diversity indices in each region are summarized (Table 1). Region A contains the highest genetic diversity (Hd = 0.842, π = 0.001667). Here G2 occurs in the highest percentage (42.3%), while G1 (19.2%), G3 (7.7%) and G5 (30.8%) occur at relatively low frequencies. In the AN region (Hd = 0.726, π = 0.000687), the central haplogroup G1 is the most abundant (45%), while in AS region (Hd = 0.881, π = 0.002177) the haplogroup G2 is the most abundant (53.1%). The lowest molecular diversity is found in region B (Hd = 0.243, π = 0.000334), where G4 (92.5%) is dominant with only a single representative of G1, G2 and G3, respectively. Region C (Hd = 0.613, π = 0.000469) is exclusively occupied by G5.

**Table 1:** Summary of numbers and frequencies of each haplogroup (G1 – G5), haplotype diversity (Hd) and nucleotide diversity (π) in the divided regions.

	AN	AS	A	B	C
G1	9 (45%)	1 (3.1%)	10 (19.2%)	1 (2.5%)	—
G2	5 (25%)	17 (53.1%)	22 (42.3%)	1 (2.5%)	—
G3	—	4 (12.5%)	4 (7.7%)	1 (2.5%)	—
G4	—	—	—	37 (92.5%)	—
G5	6 (30%)	10 (31.3%)	16 (30.8%)	—	51 (100%)
Hd	0.7263	0.881	0.8416	0.2429	0.6131
π	0.000687	0.002177	0.001667	0.000334	0.000469

AN: North Hengduan Mountains; AS: South Hengduan Mountains; A: Hengduan Mountains; B: QTP; C: north-central China, Altai and Kashmir.

**Figure 3:**
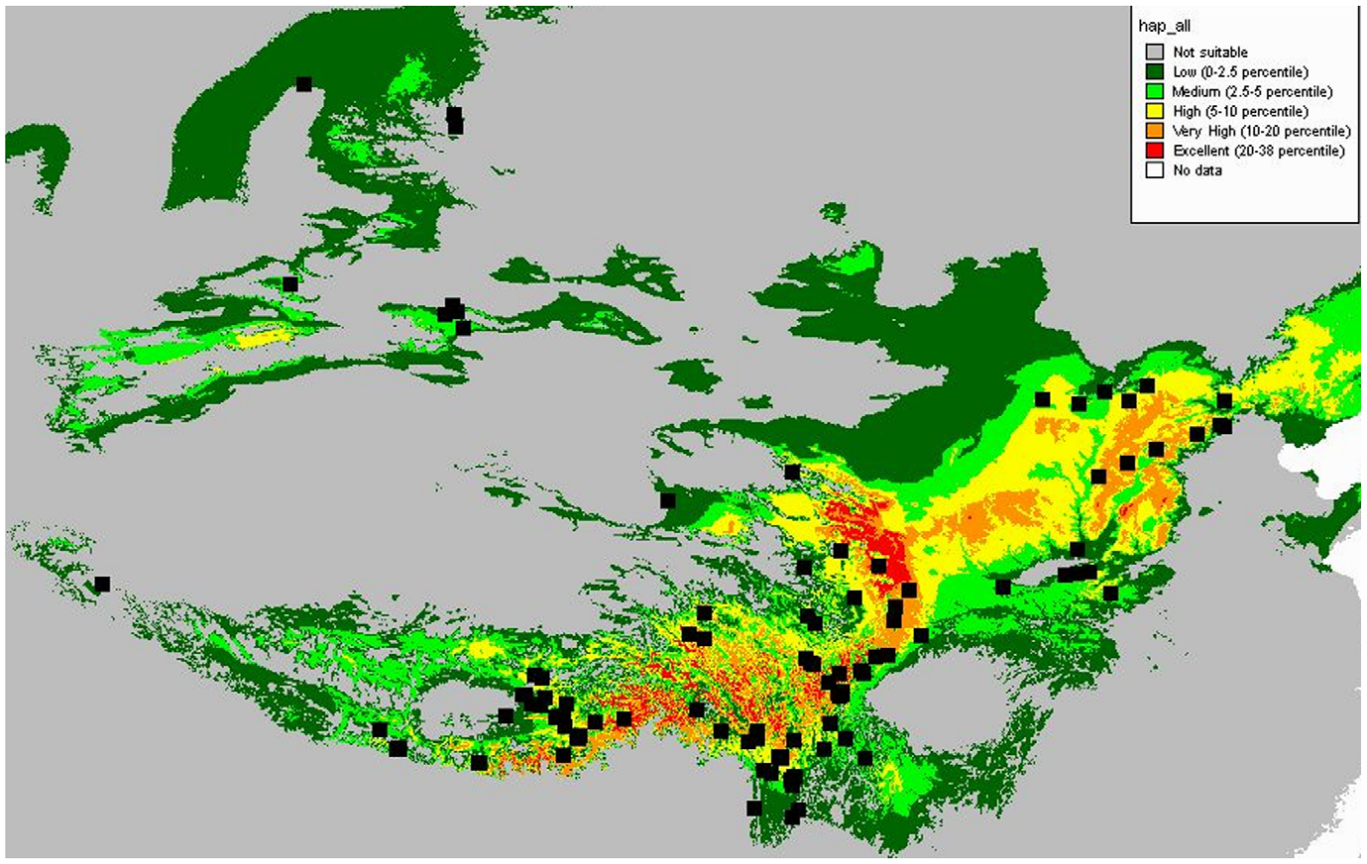
A map predicting the area of habitat potentially available for *L.clathratus*. Gradients of color are utilized to demonstrate the gradients of possibility of occurrence.

**Figure 4:**
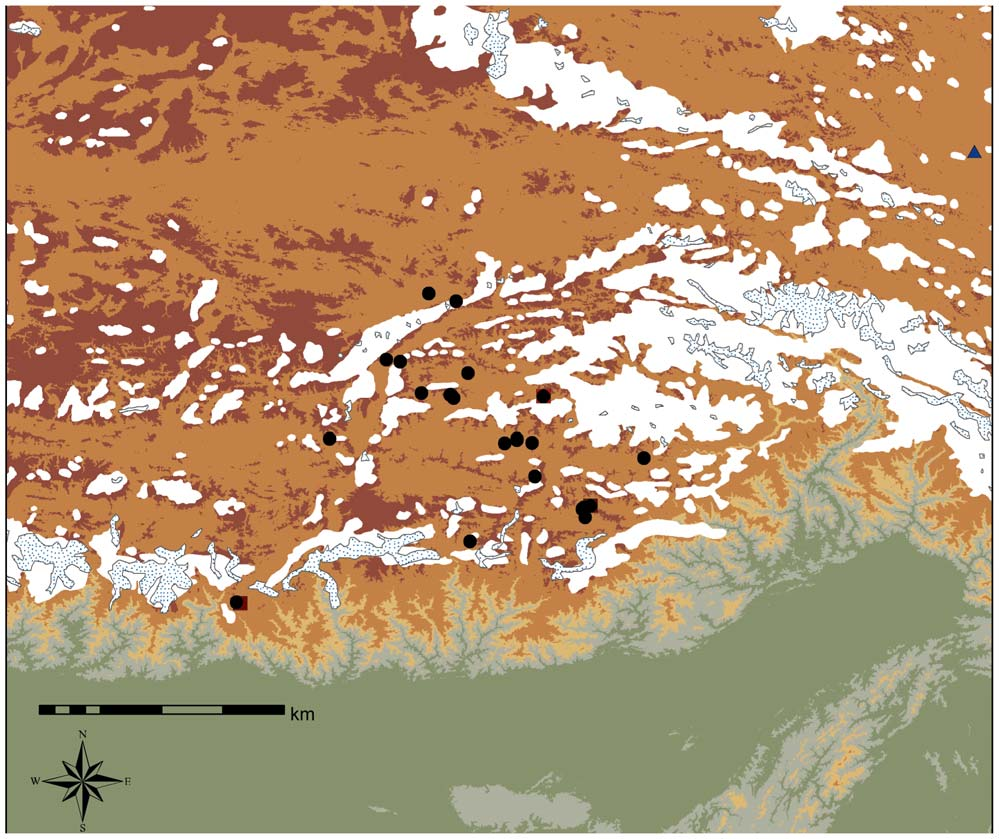
A detailed map of the QTP with the distribution of haplogroups. The white areas imply the occurrence of glaciers in LGM, while the white areas with dots indicate the occurrence of current glaciers.

The utilized ecological niche modeling module adequately predicted the distribution range of the species (Fig. 3), except for a discrepancy in southern Gansu province where relatively high probabilities of occurrence were predicted. Some populations such as the Altai population are located in areas with low probability of occurrences. The predicted occurrence in the Hengduan Mountains is highly fragmented, with high and low probability areas closely located, coinciding with the topography in this region. The detailed distribution map of the G4 haplogroup, together with LGM and current glaciers (Fig. 4), indicated that populations occur at the very rim of glaciers.

### Population structure

When we compared the genetic variation among and within populations through AMOVA, we found that a phylogeographical pattern existed between regions A and B (F_ST_ = 0.36399, P<0.001) as well as between A and C (F_ST_ = 0.25981, P<0.001). However, the spatial differentiation was relatively weak between regions AN and AS (F_ST_ = 0.06787, P = 0.01662), but after excluding the wide-spread haplogroup G5 to remove masking effect, we found a more pronounced spatial structure between AN and AS (F_S_ = 0.13963, P = 0.01369) (Table 2).

**Table 2:** Results of AMOVA analysis.

Grouping hypothesis	Va (percentage)	Vb (percentage)	F_ST_	P
1 A VS B	0.52007 (36.40%)	0.90874 (63.60%)	0.36399	0.00000
2 A VS C	0.31375 (25.98%)	0.89385 (74.02%)	0.25981	0.00000
3 AN VS AS	0.09704 (6.79%)	1.33288 (93.21%)	0.06787	0.01662
4 AN VS AS (excluding G5)	0.23163 (13.96%)	1.42723 (86.04%)	0.13963	0.01369

Region codes are the same as in Table 2. Va: variation among population; Vb: variation within population; F_ST_: fixation index.

The SAMOVA analysis showed that *F*_CT_ began to plateau for four to five groups of populations (Table 3). With four groups, the splits largely correspond to our tentative division into four regions (South Hengduan Mountains, North Hengduan Mountains, the QTP and north-central regions), except for two populations (Weixi and Jilong) assigned to North Hengduan Mountains. With five groups, the splits are the same except that Yadong and Nielamu are now removed respectively from the QTP and North Hengduan Mountains respectively to constitute a new region “Southern Himalaya”, which was also visualized by the distribution map of haplotypes. In this case, 5.48% of the variation was among populations within groups, and 42.39% of the variation was within populations. We also give the result of dividing the points into three groups (Table 3), in which North Hengduan Mountains is subsumed into the north-central regions and Southern Himalaya is not recognized.

**Table 3:** SAMOVA results that gave highest F_CT_.

	d.f.	Sum of squares	Variance components	Percentage of variation
K = 3				
Among groups	2	47.397	0.64134	45.47
Among populations within groups	26	38.65	0.22622	16.04
Within populations	98	52.213	0.54299	38.49
K = 4				
Among groups	3	61.096	0.62732	48.98
Among populations within groups	25	24.951	0.11039	8.62
Within populations	98	53.213	0.54299	42.4
K = 5				
Among groups	4	65.959	0.66769	52.13
Among populations within groups	24	20.088	0.07022	5.48
Within populations	98	53.213	0.54299	42.39

All variance components were significant (p<0.001).

### Deep history of the *L. clathratus*

**Figure 5:**
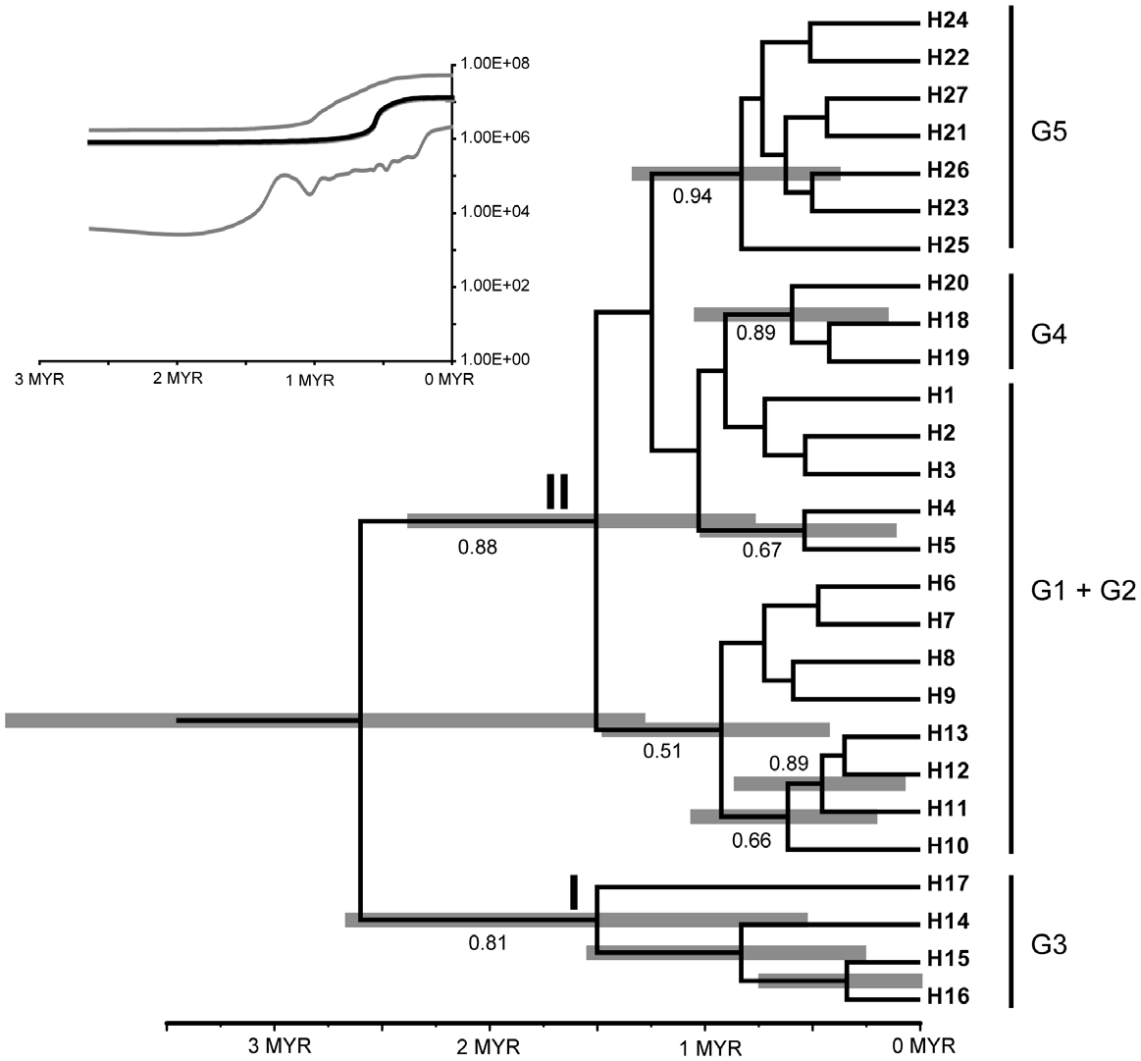
Results of coalescence analyses. Chronogram with a time scale of million years ago (Mya) shows the estimated divergence pattern assuming a constant mutation rate of 1xe-9. Grey bars reflect the length of the confidence intervals that are only calculated for nodes with a posterior support value p>0.5. These values are given above/below the branches. On the right, we provide the haplotype group classification as shown in Fig. 1. The graph inserted on the top-left of the figure shows the plot of the extended Bayesian skyline. The dark line shows the median values per time interval, whereas the grey lines show the upper and lower boundary of the 95% probability interval. The x-aches corresponds to million years in the past (Mya) whereas the y-axis corresponds to the estimated effective population sizes.

Both, maximum likelihood phylogeny and coalescence analyses recovered two distinct lineages (Fig. 5). Lineage I comprises 23 haplotypes assigned to G1, G2, G4 and G5, whereas lineage II includes only four haplotypes H14 – H17, all belonging to G3. All four haplotypes of the second lineage were found with very low frequency (total sampling 4.76%) and the statistical parsimony network suggested several extinct haplotypes connecting the recovered haplotypes (Fig. 1). Only a few clades within lineage I had some support and the haplotypes can be connected mainly by observed haplotypes. The estimated divergence times of lineage assembly suggests an accumulation of haplotypes in both lineages from around 1.5 million year ago (mya) with a confidence interval of 0.78 to 2.36 mya for lineage I and 0.55 to 2.65 mya for lineage II. Extended Bayesian Skyline analyses (Fig. 5) with two or three groups specified suggest changes in the effective population size around 0.5 to 1 mya.

## Discussion

### Recolonization of the *L. clathratus* into north-central region from its glacial refugia in the Hengduan Mountains

In the present study, range-wide genetic variation of the *L. clathratus* was surveyed using two chloroplast markers. We found that individuals from north-central China belong exclusively to the haplogroup G5, the group with the widest distribution range, whereas four out of five haplogroups occur in the Hengduan Mountains. The highly heterogeneous structure of the predicted suitable habitat in the Hengduan Mountains (Fig. 3) corresponds well with the higher genetic diversity in this area (Hd = 0.842, π = 0.001667), while the more homogeneous distribution of climatic suitability in the north-central region (Fig. 3) is linked to the lower genetic diversity there (Hd = 0.613, π = 0.000469). In addition, we detected a strong difference when comparing the genetic variation between the Hengduan Mountains region and the north-central region. The distribution pattern of haplogroups, the predicted climatic suitability, together with the result of AMOVA analysis as well as the recovered population structure in SAMOVA analysis, strongly suggest that the Hengduan Mountains provided refugia for north-central entities of the *L. clathratus* during the Quaternary glaciations. Such a “southern richness and northern purity” pattern has also been revealed by the phylogeographical study of European biota [32]. This result provided a different perspective on the origin of the north-central genetic diversity from that of a previous study of the north-central populations of *Pinus tabulaeformis* [11], in which evidence was found for a putative northern refugium, beside the Yellow River. However, we cannot exclude with the existing evidence the possibility of local survival of the blue haplogroup in areas located in northeast of the Hengduan mountians during the LGM. In this scenario, the current co-occurrences in the Hengduan Mountains may be the result of range expansion of the blue haplotype to the south-west in the Holocene. This alternative hypothesis is not consistent with the results of the Bayesian skyline analysis, which did not find a signal for a substantial transformation of the effective population size through time as required under the concept of substantial range fragmentation. However, this may be an artifact of the plastid-only data set and the result may look different in analyses considering the variation of nuclear genome. The high haplotype variation in the Hengduan mountains coincides with the fragmentation of putative occurrences caused by the complicated topography of these mountains (see Fig. 3). Future studies will need to address alternative hypotheses such as a greater impact of migration, local extinction, shift of niche preferences, and the impact of topography on the displacement of populations in responds to cooling and warming climates.

The fact that the Altai individuals, the most northerly occurrence of *L. clathratus*, harbor exclusively haplotype H21 also supports a rapid colonization of north-central China and northward to the Altai, probably accompanied with strong founder effects, which is consistent with previous findings in other plants [12]. That the geographic genetic structure of the *L. clathratus* resulted from fragmentation of geography in Hengduan Mountains and expansion in the north-central region is also consistent with the distribution of species diversity in *Lepisorus*. The HHM was determined to be not only the centre of extant species diversity but also the most likely centre of origin of the genus (Wang et al. unpublished). Furthermore, the missing/extinct haplotypes in G3 on the network (Fig. 1), which is mainly located in the HHM (Fig. 2) and represents one of the two well-split lineages in coalescence analyses (Fig. 5), is consistent with the argument for old refugia in the Hengduan Mountains and perhaps additional fragmented refugia along the southern slope of the Himalaya.

The strong differentiation within the Hengduan Mountains population(s) may be attributed mainly to the intricate topography resulting from the rapid uplift of the QTP [33]. This species topography contains numerous geographical barriers, such as lofty ridges and deep valleys, which would have greatly restricted gene flow and led to relatively independent evolution of gene pools in isolated mountain blocks. Pleistocene climate fluctuations have likely enhanced these patterns because the generally south-north directions of the valleys may have enforced not only migrations along altitudinal gradients but also along several independent south-north gradients. This hypothesis is also supported by the high population differentiation documented by cytoplasmic DNA markers in several plants distributed in this region [15], [34].

It is very likely that the recolonization of the *L. clathratus* along the mountain ranges in the north-central region from the Hengduan Mountains has been achieved through dispersal events (G5 blue haplogroup). Entities in north-central regions all have dehiscent sporangia leading to unimpaired dispersal ability, which accounts for the wide distribution of the haplogroup G5. It is likely that reduction of genetic diversity (bottlenecks) likely occurred along the migration route since only a single haplogroup was detected for the north-central region populations. Reduced allele diversity, together with genetic homogeneity, were expected for populations in the area occupied by interglacial expansion, because it would be much more difficult for those behind the pioneers to advance when the area was swept by the Quaternary glaciations and recolonized rapidly by leading edge populations from the borders of refugia [6].

The SAMOVA analysis revealed a distinction between the North and South Hengduan Mountains, which can be seen on the distribution map of haplogroups (Fig. 2). This population differentiation is also detected in AMOVA analyses, although it is a weak differentiation (F_ST_ = 0.06787) but with a more marked pattern of population differentiation (F_ST_ = 0.13963) after excluding the masking effect of the wide-spread haplogroup G5. Between the two sub-regions, South Hengduan Mountains possesses a higher genetic diversity (Table 1), which is also consistent with the SAMOVA results, i.e., South Hengduan Mountains precedes North Hengduan Mountains as a recognizably separate population group. The distinction is also visible in the results of the coalescence analyses (Fig. 5), in which H1 – H5 are mainly represented by Sichuan and Tibetan specimens, while H6 – H13 are dominated by Yunnan specimens. The spatial distinction is also found in a previous study [15], [35] and the Western Sichuan Plateau may serve as a barrier to gene flow between northern and southern Hengduan Mountains. Further study with denser sampling will aid exploration of the underlying phylogeographical structure.

### Phylogeographical history of the *L. clathratus* in the QTP regions

Although each species has its own unique history during glacial and interglacial periods, pioneer phylogeographical studies of alpine plants in the QTP and HHM regions have revealed two widespread patterns (described as above). Our study reveals the phylogeographical history of alpine ferns occurring in this area for the first time, improving our understanding of phylogeographical patterns of vascular plants in the QTP and HHM.

Haplotype H1 and the rare haplotypes H2 – H13 are mostly distributed in the Hengduan Mountains, while the overwhelming majority of individuals with indehiscent sporangia bear H18 and its two derivatives H19 and H20, which are only found on the QTP — the sole exception occurring in the southern Himalaya (Yadong). At the same time, two representatives carrying ancestral haplotype H1 and the rare haplotype H2 were detected on the QTP. Moreover, the AMOVA analysis detected a strong phylogeographical structure between the QTP and the Hengduan Mountains. This evidence implies that the populations with non-shared haplogroup G4 most likely survived on the QTP in situ during the Quaternary glaciations and the low frequent haplotype H1 and H2 may have been the result of long-distance dispersal from individuals in the Hengduan Mountains during postglacial periods. The still existing glaciers between region A and B (Fig. 1) might be an orographical barrier that explains the phylogeographical differentiation between these two regions.

In previous studies, some plants in the QTP have been found to possess low genetic diversity and derived haplotypes, as expected for areas that were re-colonized after the LGM from the eastern or southeastern refugia [9], [10], [12], [15]. In an alternative scenario, plants in the QTP show high genetic diversity and ancestral haplotypes, which suggest an in situ survival during the LGM on the plateau [13], [18]. Nevertheless, our study reveals a distinct phylogeographical history of plants occurring in the QTP, which has not been reported before. Even though populations of the *L. clathratus* on the QTP possess lower genetic diversity (Table 1) and derived haplotypes, they most likely survived in situ during the LGM, as shown by an abundance of derived haplotypes that are endemic to this region. The result of coalescence analysis provides further evidence for this argument. The resultant diversification of G4 haplotypes (private haplotypes on the QTP) dates back to around 0.5 Myr ago (Fig. 5), which is well before the LGM (about 20,000 years ago). Therefore, individuals with the private haplotype may have shifted to the microrefugia during LGM and pre-LGM glaciations and expanded to the nearby areas afterwards.

The geographical status of the QTP in the Quaternary endows our argument with a reasonable explanation. Currently, it is generally accepted that there was not a unified ice sheet covering the QTP, at least not during the last few glacial cycles of the Quaternary [36], [37], and advances of the Tibetan glaciers were much less prominent than elsewhere in the northern hemisphere, most likely due to arid conditions and high sublimation rates [38]. Therefore, potential habitats for cold-tolerant species could be found in the plateau in that period. Recent phylogeographical studies suggested that *Aconitum gymnandrum* [13] and *Potentilla fruticosa* [18] may have survived the more recent or all quaternary glaciations on the plateau. *L. clathratus* is likely another case of such relatively cold-tolerant plants. Even though these plants are deciduous during the winter, the underground rhizomes stay alive through the dry and cold period, and the new leaves emerge from the dormant rhizome in the following year. Furthermore, the restricted distribution of G4 haplotypes (Fig.4) together with the distribution of current glaciers and LGM explicitly indicates that entities with indehiscent sporangia can occur at the very rim of glaciers. This evidence points to a strong cold-tolerant ability in these alpine ferns. Therefore, the distribution of individuals with indehiscent sporangia in *L. clathratus* may contract to the non-glaciated regions during Quaternary glaciations and expand to larger areas during interglacial periods.

Two questions are still required to be considered. How do the entities with indehiscent sporangia keep their remarkable genetic homogeneity and why is their distribution range tend to be restricted? The explanation is likely given by the characteristics of the sporangium of these individuals. Specimens with G4 (H18 - H20) haplotypes possess exclusively indehiscent sporangia, which is unusual in ferns. Previous studies [39], [40] indicate that sporangial dehiscence happens owing to the differential thickening of annular cells. Their inner and radial walls are thickened, but the outer and side walls remain thin. When the annulus arches backwards because of cell contraction, it exerts pressure on the elongated and thin walled cells of the stomium. Eventually sufficient pressure is developed to cause rupture of the stomium and wide open of the sporangium. However, individuals with indehiscent sporangia, which used to be regarded as an independent genus *Platygyria* [28], are identified by a very broad annulus consisting of all non-thickened cell-walls and indistinct stomium. The uniformity of annular cells leads to the failure of the annulus to tear. In this case, the sporangium, with its complete spore-content, becomes the unit of dispersal and mature spores are retained within the sporangium until the decay of the sporangial wall. On one hand, it has a negative effect upon the species’ capacity for dispersal because a great proportion of spores are retained in the immediate vicinity of the parent populations. This reproduction mode will likely result in restricted distribution ranges. Brownsey [40] pointed out that the reduced capacity of dispersal is considered to be adaptively advantageous for a species which occupies a highly specialized chasmophytic habitat and whose populations are often separated by large distances. Individuals with indehiscent sporangia in *L. clathratus* are restricted to crevices of natural stone outcrops on the damper sides of steep mountains. Therefore, the reduced dispersal capacity may be evolutionarily beneficial for their survival and coincide with the hypothesis of long-term survival of populations more or less in-situ through glaciations. On the other hand, the retention of spores in the sporangium naturally adds the possibility of coherence of spores, known as “synaptospory” [41], which may reduce the possibility of outcrossing but increase the probability of intergametophytic selfing. Though most species of homosporous ferns would be classified as outcrossers with only a few being nearly exclusively inbreeding [42], we can see a great likelihood of intergametophytic selfing in the G4 lineage, which favors maintaining genetic homogeneity.

Entities with indehiscent sporangia are scattered across the haplotype network (Fig. 1). This pattern may be explained either as the result of independent origins of a mutation causing the formation of indehiscent sporangia, or the spread of the character via recombination through the populations of *L. clathratus*. To understand the later pattern, we need to consider that plants with indehiscent sporangia are dominantly intergametophytic selfers, but still have the potential to contribute sperm cells to the reproduction of outcrossing gametophytes that are generated from spores of dehiscent sporangia [43]. The resulting reproductive pattern may be comparable to a mixed mating population, containing inbreeders and outbreeders resulting in transfer of haplotypes among sporangium form. Sporangial indehiscence has been shown to be a poor indicator of specific delimitation besides generic delimitation [31]. The further exploration of these hypotheses requires at least the reconstruction of the relationships using evidences of nuclear genome variation and crossing experiments.

One may argue that the private haplogroup on the QTP could be the result of colonization of haplotypes from southern Himalaya. The result of SAMOVA analysis suggested the South Himalaya as a possible refugium for individuals with uncommon sporangial pattern in *L. clathratus*. This is congruent with the results of a recent phylogeographical study of *Juniperus tibetica* complex [17], which illustrated the potential significance of high-mountain areas for glacial microrefugia. The large altitudinal gradients in the Himalaya resulted in a large climatic buffer (0.55°C/100m) [44]. Plants may alter their altitudinal range (phalanx migration) in response to shifts in optimal habitats during the Quaternary climatic oscillation. Nevertheless, the sampling in the region is so limited that we cannot draw any conclusions, and an increase of sampling coverage and density in this region would help to clarify the hidden phylogeographical pattern.

Further comparative studies with other regions and other species are required to confirm the history of alpine species occurring in the QTP-HHM. Extensive studies conducted in high-altitude regions will allow us to extract shared phenomena among species, although each species has its own unique history during glacial and interglacial periods [45].

## Materials and Methods

### Ethics statement

This study did not require any special permits because all collecting was performed by researchers located at institutes with the permits required such as IBCAS (Institute of Botany, Chinese Academy of Sciences) in Beijing.

### Plant collection and species concept

A total of 147 individuals (Table S1) were sampled throughout the mainland Asia range of *L. clathratus*, extending from the QTP and the HHM, to northern China and the Altai mountain ranges in Russia and westward to Nepal and Kashmir along the southern slope of the Himalaya. The sampling of these alpine plants was difficult since individuals were scarce and scattered, and usually found on mountain tops or on the damper and cooler faces of steep and high mountain ranges. Our sampling is particularly limited along the southern edge of the Himalaya. Populations usually consist of a few individuals, and suitable areas for colonization are often separated by long distances. The long creeping rhizomes of these ferns may result in the formation of clones as a result of individuals multiplying through fragmentation of the rhizome. Therefore, at each site we collected as many leaves as possible from spatially separated individuals. The leaf material was dried in silica gel and stored at room temperature.

We employed a broad species concept for *L. clathratus*, that is, treated the whole complex as one single species [27]–[29], [31]. *Platygyria* was nested within the complex and the sporangial dehiscence/indehiscence was considered as an intraspecific trait. Currently, we do not have any evidence for hybridization in the complex. More exhaustive analyses concerning the species delimitation in the complex are currently being performed and will be reported elsewhere. Our sampling includes several segregates including those recognized in the most recent revision of the complex: *Lepisorus albterii* (Regel) Ching, *L. crassipes* Ching & Y. X. Lin, *L. likiangensis* Ching & S. K. Wu, and *L. thaipaiensis* Ching & S. K. Wu [25]. Our results support their treatment as a synonym of *Lepisorus clathratus* as suggested by some authors [27]–[29] but additional evidence is required and will be obtained in ongoing studies.

### DNA extraction, amplification and sequencing

Total genomic DNA was extracted from silica gel dried leaves using the modified CTAB procedure of Doyle and Doyle [46]. For each individual, two plastid genome regions (*trn*L-F region including the *trn*L intron + the *trn*L*-trn*F intergenic spacer (IGS), and the *rps*4-*trnS* region including *rps*4 + *rps*4*-trn*S IGS) were amplified separately with standard polymerase chain reaction (PCR) conditions and with previously published primer sets [47]–[50]. The PCR products were purified using GFXTM PCR DNA and Gel Band Purification kits (Amersham Pharmacia Biotech, Piscataway, NJ, USA), and were then directly sequenced. Sequencing reactions were conducted using the DYEnamicTM ETDye Terminator Cycle Sequencing Kit (Amersham Pharmacia Biotech). All sequences have been deposited at GenBank (see Table S2 for accession numbers).

Sequences were analyzed using MegaBACETM1000 DNA Analysis Systems, following the manufacture’s protocols. Sequence data were edited and assembled in ContigExpress program from the Vector NTI Suite 6.0 (Informax Inc., North Bethesda, MD). The resulting sequences were aligned using CLUSTAL X [51], further adjusted manually in BioEdit [52] and MacClade 4.08 [53] and indel changes were excluded. Ambiguous positions were detected visually and excluded for the following analyses. For a conservative understanding of dataset, we excluded all indels.

### Assessing relationships of haplotypes

Phylogenetic relationships of the recovered haplotypes were reconstructed using algorithms that create networks assuming the occurrence of ancestral haplotypes, such as minimum spanning trees, as implemented in the software Splitstree 4.10 [54] and as median networks by statistical parsimony [55], as implemented in TCS 1.21 [56]. These networks were also visualized together with the frequency of the observed haplotypes and the occurrence of sporangium types (dehiscent versus indehiscent).

### Assessing spatial genetic differentiation

To produce distribution maps of the studied species, we used ArcGIS 9.3 [57]. The SRTM elevation dataset (used as a background elevation map) was downloaded from WorldClim website (http://www.worldclim.org). The area of the habitat potentially available for *L. clathratus* was modeled in the DIVA-GIS 7.1.7.2 [58] environment using an Ecological Niche Modeling module with all 19 climatic variables and output type Bioclim.

To date, there is no consensus regarding the glaciations of the QTP at the LGM. Based on the interpretation by Kuhle [59] of glacial geology, the ice sheet could have been up to 2 km thick and covered the entire plateau at elevations over 3,000 m. Here, we show the minimum-glaciation range reconstruction based on the interpretation by Ehlers and Gibbard [60] of glacial geology.

In order to investigate genetic variation in different regions, we tentatively divided the sampling into three genetically similar regions according to the distribution of haplotype groups (Fig. 2): Hengduan Mountains (A), the QTP (B) and north-central China plus the Altai and Kashmir (C). As the sampling within the southern Himalaya was insufficient to consider a deeper investigation, we currently deliberately overlook this region. Region A is divided into two sub-regions, South Hengduan Mountains (AS) and North Hengduan Mountains (AN), which is part of the western Sichuan Plateau. Molecular diversity indices, including haplotype diversity (Hd) and nucleotide diversity (π), were estimated for each region using Arlequin 3.11 [61].

### Testing population structure

Analyses of molecular variance (AMOVA) [62] were performed to assess genetic differentiation within populations and between populations within groups using the program Arlequin 3.1 [61] with significance test based on 1,000 permutations and hapolotype pair-wise genetic distances incorporated.

To test whether the pre-defined regions are consistent with genetically differentiated population groups, we analyzed our data with a spatial analysis of molecular variance (SAMOVA). Populations with only one representative were deleted from this analysis. Geographically close populations were clustered into a user-defined number of groups (K) using a simulated annealing approach to maximize the variance (*F*_CT_) among those groups [63]. The analysis was constructed in SAMOVA 1.0 [63] using K = 2–10, and we chose the number of groups for which *F*_CT_ began to plateau.

### Inferring the deep history of the *L. clathratus*

The pre-LGM history of the species was inferred by reconstructing the phylogeny of the lineage using maximum likelihood optimization as implemented in GARLI 0.951 [64]. Each haplotype was represented with a single specimen and we incorporated two species as outgroups. The outgroup selection was based on the results of a previous phylogenetic study [31] and the model of substitution evolution was determined using jModeltest 0.1 [65]. We also inferred the deeper relationships by using a coalescence analytical approach as implemented in BEAST 1.4. (http://beast.bio.ed.ac.uk/). We initially used several mutation rates reported for land plants [66], but the final analyses were done with a constant mutation rate of 1.0 e-9, as the obtained time frame was consistent with the divergence time estimate of the crown group of the *L. clathratus* clade in an independent divergence time estimation for the whole genus (Wang et al. unpublished). These analyses were performed with a dataset including a single representative of each haplotype without outgroup taxa. Several models of coalescence analyses were performed, including Bayesian Skyline under step-wise constant population sizes with two to 10 groups and the Extended Bayesian Skyline that does not require the number of groups to be specified, but allows to integration of the information in the inheritance mechanisms of the markers [67], [68]. The results of each analysis were compared with others using Tracer 1.5 (http://tree.bio.ed.ac.uk/software/tracer) and by visualizing the chronograms in Figtree 1.3.1. (http://tree.bio.ed.ac.uk/software/figtree). Bayesfactor analyses carried out using Tracer suggested a scenario of two or three lineages as likely to best fit the observed data. The coalescence analyses provided also estimates of the effective population size through time.

## Supporting Information

Table S1 **Locality, sample sizes (N), cpDNA haplotypes and its numbers, haplogroup and coordinate of the studied** ***L. clathratus*** **populations.**
(DOC)

Table S2 **GenBank accession numbers for haplotype sequences included in the phylogeographical analyses.**
(DOC)
