# Supplementary material for: Phylogeography of the Sino-Himalayan Fern Lepisorus clathratus on “The Roof of the World”
Source: PLoS One. 2011 Sep 30;6(9):e25896. doi: 10.1371/journal.pone.0025896 (PMC3184171; doi:10.1371/journal.pone.0025896)
Supplement: Table S1 — Locality, sample sizes (N), cpDNA haplotypes and its numbers, haplogroup and coordinate of the studied L. clathratus populations. (DOC) [file pone.0025896.s001.doc]

Table S1 Locality, sample sizes (N), cpDNA haplotypes and its numbers, haplogroup and coordinate of the studied *L. clathratus* populations.

| No. | Locality | N | Haplotypes (numbers) | Haplogroup | Longititude | Latitude |
| --- | --- | --- | --- | --- | --- | --- |
| South of Hengduan Mt. |  | | | | | |
| 1 | Cang Mt., Yunnan | 3 | H11(1), H15(2) | G2 G3 | 100.1648 | 25.6459 |
| 2 | Binchuan, Yunnan | 2 | H11(1), H13(1) | G2 | 100.4075 | 25.9417 |
| 3 | Lushui, Yunnan | 1 | H15(1) | G3 | 98.8167 | 25.9667 |
| 4 | Lijiang，Yunnan | 7 | H1(4), H4(1), H6(1), H21(1) | G1, G2, G5 | 100.2617 | 27.0950 |
| 5 | Weixi，Yunnan | 3 | H8(1), H21(1), H27(1) | G2, G5 | 99.3379 | 27.2603 |
| 6 | Butuo，Sichuan | 3 | H21(3) | G5 | 102.8156 | 27.7516 |
| 7 | Shangrila，Yunnan | 8 | H1(1), H7(1), H11(2), H12(1), H21(3) | G1, G2, G5 | 99.7647 | 27.8131 |
| 8 | Muli，Sichuan | 1 | H4(1) | G2 | 101.3269 | 28.1204 |
| 9 | Deqin，Yunnan | 1 | H9(1) | G2 | 98.9099 | 28.7677 |
| 10 | Chayu，Tibet | 1 | H15(1) | G3 | 97.6122 | 28.7850 |
| 11 | Daocheng，Sichuan | 1 | H10(1) | G2 | 100.3052 | 29.1282 |
| 12 | Ranwu，Tibet | 1 | H21(1) | G5 | 96.7545 | 29.5045 |
| North of Hengduan Mt. |  | | | | | |
| 13 | Jiulong，Sichuan | 2 | H1(2) | G1 | 101.5330 | 29.0167 |
| 14 | Kangding，Sichuan | 5 | H1(2), H8(1), H21(2) | G1, G2, G5 | 101.9014 | 30.0731 |
| 15 | Danba，Sichuan | 2 | H1(2) | G1 | 101.6785 | 30.6731 |
| 16 | Baoxing，Sichuan | 2 | H1(1), H21(1) | G1, G5 | 102.6856 | 30.8726 |
| 17 | Luhuo，Sichuan | 7 | H1(2), H4(4), H21(1) | G1, G2, G5 | 100.7398 | 31.3089 |
| 18 | Lixian，Sichuan | 1 | H26(1) | G5 | 103.1667 | 31.4167 |
| 19 | Wenchuan，Sichuan | 1 | H21(1) | G5 | 103.6167 | 31.4667 |
| the QTP |  | | | | | |
| 20 | Naidong，Tibet | 13 | H18(13) | G4 | 91.8195 | 29.1256 |
| 21 | Langxian，Tibet | 1 | H18(1) | G4 | 93.0734 | 29.0898 |
| 22 | Sangri，Tibet | 1 | H18(1) | G4 | 91.9226 | 29.2433 |
| 23 | Renbu，Tibet | 1 | H18(1) | G4 | 89.8407 | 29.2864 |
| 24 | Muzhugongka，Tibet | 3 | H1(1), H18(2) | G1, G4 | 92.0403 | 29.7156 |
| 25 | Lasha，Tibet | 9 | H2(1), H18(7), H20(1) | G2, G4 | 91.0935 | 29.7804 |
| 26 | Longzi，Tibet | 6 | H17(1), H18(4), H19(1) | G3, G4 | 92.4800 | 28.5700 |
| 27 | Yangbajing，Tibet | 3 | H18(3) | G4 | 90.5211 | 30.0814 |
| 28 | Dangxiong，Tibet | 2 | H18(2) | G4 | 91.0028 | 30.7351 |
| North-central China, Altai, Kashmir |  | | | | | |
| 29 | Pingwu, Sichuan | 1 | H21(1) | G5 | 104.8265 | 32.1923 |
| 30 | Songpan, Sichuan | 2 | H21(2) | G5 | 103.8235 | 32.7529 |
| 31 | Jiuzhaigou, Sichuan | 1 | H21(1) | G5 | 103.9135 | 33.2278 |
| 32 | Luanchuan, Henan | 5 | H27(5) | G5 | 111.6460 | 33.7358 |
| 33 | Zhouqu, Gansu | 1 | H21(1) | G5 | 104.3833 | 33.8167 |
| 34 | Taibai Mt., Shaanxi | 7 | H21(3), H23(1), H24(3) | G5 | 107.7635 | 33.9662 |
| 35 | Kashmir, India | 1 | H22(1) | G5 | 75.2951 | 34.0564 |
| 36 | Qinghai | 7 | H21(6), H23(1) | G5 | 100.5737 | 34.1626 |
| 37 | Lingbao, Henan | 4 | H21(2), H23(1), H24(1) | G5 | 110.5841 | 34.6731 |
| 38 | Xian, Shaanxi | 1 | H27(1) | G5 | 95.6771 | 37.0625 |
| 39 | Lvliang, Shanxi | 1 | H22(1) | G5 | 111.2421 | 37.9377 |
| 40 | Lumao Mt., Shanxi | 1 | H22(1) | G5 | 112.2473 | 38.4170 |
| 41 | Xiaowutai Mt., Hebei | 1 | H25(1) | G5 | 114.7632 | 39.4828 |
| 42 | Beijing | 2 | H21(2) | G5 | 115.6606 | 39.7732 |
| 43 | Dahaituo, Hebei | 1 | H21(1) | G5 | 115.7315 | 40.6755 |
| 44 | Inner Mongolia | 6 | H21(3), H22(3) | G5 | 111.3086 | 40.8648 |
| 45 | Wulumuqi, Xinjiang | 2 | H21(2) | G5 | 87.8951 | 43.8404 |
| 46 | Altai Mt，Russia | 6 | H21(6) | G5 | 87.1072 | 51.1200 |
| Southern Himalaya |  |  |  |  |  |  |
| 47 | Yadong，Tibet | 2 | H16(1), H18(1) | G3, G4 | 88.9000 | 27.6000 |
| 48 | Nielamu，Tibet | 2 | H5(1), H14(1) | G2, G3 | 85.9900 | 28.1300 |
| 49 | Jilong，Tibet | 2 | H3(1), H6(1) | G2 | 85.2979 | 28.7965 |
