# Supplementary material for: Phylogeography of the Sino-Himalayan Fern Lepisorus clathratus on “The Roof of the World”
Source: PLoS One. 2011 Sep 30;6(9):e25896. doi: 10.1371/journal.pone.0025896 (PMC3184171; doi:10.1371/journal.pone.0025896)
Supplement: Table S2 — GenBank accession numbers for haplotype sequences included in the phylogeographical analyses. (DOC) [file pone.0025896.s002.doc]

Table S2 GenBank accession numbers for haplotype sequences included in the phylogeographical analyses.

| **haplotype** | ***rps*4-*trn*S** | ***trn*L-F** |
| --- | --- | --- |
| **H1** | JF938566 | JF938539 |
| **H2** | JF938567 | JF938540 |
| **H3** | JF938568 | JF938541 |
| **H4** | JF938569 | JF938542 |
| **H5** | JF938570 | JF938543 |
| **H6** | JF938571 | JF938544 |
| **H7** | JF938572 | JF938545 |
| **H8** | JF938573 | JF938546 |
| **H9** | JF938574 | JF938547 |
| **H10** | JF938575 | JF938548 |
| **H111** | JF938576 | JF938549 |
| **H12** | JF938577 | JF938550 |
| **H13** | JF938578 | JF938551 |
| **H14** | JF938579 | JF938552 |
| **H15** | JF938580 | JF938553 |
| **H16** | JF938581 | JF938554 |
| **H17** | JF938582 | JF938555 |
| **H18** | JF938583 | JF938556 |
| **H19** | JF938584 | JF938557 |
| **H20** | JF938585 | JF938558 |
| **H21** | JF938586 | JF938559 |
| **H22** | JF938587 | JF938560 |
| **H23** | JF938588 | JF938561 |
| **H24** | JF938589 | JF938562 |
| **H25** | JF938590 | JF938563 |
| **H26** | JF938591 | JF938564 |
| **H27** | JF938592 | JF938565 |
